# Bipolar Disorder Patients Follow-up (BDPF): methods and materials

**Published:** 2010

**Authors:** Amir Shabani, Aida Taheri, Sanaz Azadforouz, Chehreh Najd Abbasi, Zahra Mousavi, Kambiz Zangeneh, Seyed Vahid Shariat, Shabnam Nohesara, Morteza Naserbakht, Mojgan Taban, Sadaf Kokar, Samaneh Teimoorinejad, Mitra Hakimshooshtary, Elham Shirazi

**Affiliations:** a Bipolar Disorders Research Group, Mental Health Research Centre, Iran University of Medical Sciences and Health Services, Tehran, Iran; b Tehran Psychiatric Institute, Iran University of Medical Sciences and Health Services, Tehran, Iran; c Tabriz University of Medical Sciences and Health Services, Tabriz, Iran; d Iran Hospital of Psychiatry, Iran University of Medical Sciences and Health Services, Tehran, Iran

**Keywords:** Bipolar Disorder, Follow Up, Naturalistic Study, Outcome Study, Longitudinal Study, Iran

## Abstract

**BACKGROUND:**

The Bipolar Disorder Patients Follow-up (BDPF) project is a longitudinal, prospective and naturalistic study. The purpose of the present report is to introduce the project, elaborate its methods, and present the reliability data of the utilized symptoms rating scales.

**METHODS:**

The sampling started in May 2008 and is still in progress. The probands are assessed at the beginning of the sampling and then 2 and 6 months later and then every 6 months using several instruments to identify psychiatric comorbidities, symptoms severity, quality of life, attempted suicide rate, treatment compliance, and some other factors.

**RESULTS:**

The results could lead to increase the clinicians’ awareness about the clinical picture of this disorder in Iranian patients.

**CONCLUSIONS:**

The present project could decrease to some extent the current shortcomings in Iran’s psychiatric data at least about one of the major psychiatric disorders known as the eighth result which cause medical disability over the world.

The Bipolar Disorder Patients Follow-up (BDPF) project is a prospective and longitudinal study on the patients with bipolar disorder (BD) type I who have been hospitalized in Iran Hospital of Psychiatry, a large mental hospital located in the metropolitan city of Tehran, Iran. The Mental Health Research Centre funded the BDPF. The project aimed at presenting a naturalistic clinical picture of BD type I in part of Iran. The purpose of the present report is to introduce the project, elaborate its methods, and present the reliability data of the utilized symptoms rating scales.

Bipolar disorder is a prevalent psychiatric disorder with the properties of recurrent mood episodes, high rate of suicidality, as well as a significant burden on the society and considerable economic costs. Although the issue of BD has recently received much attention, the researchers in Iran have not yet shown adequate interest toward the subject. Based on “a scientometric analysis of studies on mood disorders in Iran^1^”, only 75 articles about BD were published until January 2008.

Recently, a cohort study was carried out on bipolar patients to assess the outcome of the BD type I in Roozbeh Hospital in Tehran.^2^,^3^ The patients (n = 131) were followed for one year and the study did not continue. To the best of our knowledge, only one other longterm follow-up study has been performed on patients with BD in Iran.^4^,^5^,^6^ This study was on the patients with BD type I referred to Iran Hospital of Psychiatry, Tehran, who suffered from their first manic or mixed mood episode. They were followed for 8 to 24 months but the sample size was small (n = 23).

Apart from two mentioned longitudinal investigations, a retrospective assessment on the course features of BD type I in Tehran has recently been reported.^7^,^8^ Furthermore, there is a paper from Isfahan, Iran, that reports the relapse/recurrence rate of BD during a year in patients hospitalized in an emergency ward without identifying the type of BD.^9^

As previously noticed, the evidence on the natural course, outcome and burden of BD in Iran is scarce. Therefore, performing a longterm well-designed naturalistic study with measuring various aspects of BD is necessary.

## Methods

The BDPF is a prospective, longitudinal and naturalistic study in which the patients hospitalized in one ward of women and two wards of men in the Iran Hospital of Psychiatry, Tehran, Iran, are enrolled and followed. This mental hospital admits the patients with psychiatric disorders from over the country without any limitation in admitting the patients with different psychiatric diagnoses.

### 

#### Sampling and Participants

All the patients who are admitted to the beds supervised by the authors (SN, AS, and SVS) and some other attends of psychiatry in the hospital are assessed concerning the inclusion and exclusion criteria. Therefore, the sampling will be sequential.

The inclusion criteria are: being aged 18 years or higher, being able to speak Persian, living in Tehran, Karaj or suburbs, being diagnosed as BD type I by a board certified psychiatrist based on the Diagnostic and Statistical Manual of Mental Disorders, 4^th^ edition, Text Revision (DSM-IV-TR)^10^ and taking the same diagnosis with the Structured Clinical Interview for DSM-IV axis I disorders (SCID-I) performed by a trained resident of psychiatry, having at least one telephone line as well as one cell phone to facilitate the contact and giving informed written consent. The exclusion criterion is being mentally retarded or having any other permanent cognitive decline.

#### Procedure

At first, the residents of psychiatry were trained to perform the SCID-I and other measurements. After the training, they did the SCID-I on several patients and finally the interrater reliability was calculated.

The eligibility of the patients to enter the study is assessed by the residents of psychiatry based on a fixed schedule. A coordinator introduces every eligible patient to the residents according to their turn. Every resident carries out all measurements on her/his probands and follows them up. The residents invite the probands to continue their treatment with coming to the outpatient clinic of the hospital in a regular basis and refer to the follower physician. The treatment is not based on any fixed program or a common algorithm; instead, it is performed routinely and according to the everyday practice of the physicians. However, if the patients do not agree to be referred to the same resident for continuing the treatment, the patient is referred to another physician for treatment but the follow up measurements are performed according to the predetermined schedule by the referring resident. The research coordinator arranges the follow up sessions for the patients. Whenever the patients’ attendance at the outpatient clinic of the hospital would not be possible on the scheduled date, a home visit is planned. If the patient or her/his family does not allow for a home visit, a telephone follow up will be the final way.

The sampling started in May 2008 and is still going on. The probands are evaluated at the beginning of the study and 2 and 6 months later and every 6 months thereafter. The following instruments are utilized for the evaluations in the visits.

#### Instruments

1. ***Demographic and Clinical Variables Questionnaire:*** Demographic characteristics and certain clinical features like the information regarding previous hospitalizations and major mood episodes, history of treatments, and adherence to medications, were included in this questionnaire.
2. ***The Structured Clinical Interview for DSM-IV axis I disorders (SCID-I)^11^,^12^,^13^:*** The SCID-I is a semistructured interview to diagnose the axis I disorders of DSM-IV.^11^ The Persian clinical version of the SCID-I has been standardized for the Iranian popula-tion.^13^
3. ***Hamilton Depressive Rating Scale-7 (HDRS-7)^14^:*** HDRS is widely used in psychiatric research with favorable reliability and validity^15^ even in telephone interview.^16^ McIntyre et al^14^ reported that the 7-item HDRS had as much effectiveness as the 17-item one. The total score of HDRS-7 ranges from 0 to 26.
4. ***Young-Mania Rating Scale (Y-MRS)^17^:*** The Y-MRS is a clinician (or trained rater) administered scale to measure the severity of manic symptoms and includes 11 items with the total score that ranges from 0 to 60. A psychometric evaluation of its Persian version demonstrated a favorable reliability and validity of the scale.^18^
5. ***Scale for the Assessment of Positive Symptoms (SAPS)^19^,^20^:*** The SAPS is a clinician (or trained rater) administered scale to measure the severity of psychotic symptoms and includes 35 items in 5 sections. Favorable psychometric values for SAPS administered on an Iranian population were reported.^21^
6. ***Clinical Global Impression (CGI)^22^:*** It rates the patient’s function quickly by clinician and simply shows a general picture of the current state.^22^ It includes three items: severity of illness, global improvement and efficacy index.
7. ***Global Assessment of Functioning (GAF) scale^10^:*** The GAF scale is to measure the psychosocial function of patients with psychiatric disorders. It is used during a clinical interview and its score ranges from 0 to 100.^10^ The intra-class correlation coefficient of 0.58 was reported on a sample of bipolar patients in Iran.^2^
8. ***The World Health Organization Quality of Life (WHOQOL)-BREF^23^:*** This is a self-report questionnaire with 26 items including four domains that are used to assess the patients’ quality of life. Each item is scored from 0 to 5. Its Persian version has been provided on behalf of the World Health Organization.^23^ A favorable reliability for the Persian version has been reported on an Iranian clinical sample.^3^
9. ***Drug Attitude Inventory-10 (DAI-10) (Shortened Version)***^24^: It is a self-report inventory to rate the attitude of patients toward the prescribed medications and the effects of this attitude on adherence to the medications.^24^ It has already used on Iranian bipolar patients.^25^
10. ***Family Interview for Genetic Studies (FIGS)^26^:*** The FIGS is an interview to determine psychiatric disorders in the family. The interview includes general screening questions and a few checklists to consider each diagnosis specifically.^26^ The present study just utilizes the General Screening questions, the Depression Checklist and the Mania Checklist.

## Results

### 

#### Reliability of The Symptoms Rating Scales

The inter-rater reliability of the HDRS-7, YMRS and SAPS were evaluated in this study. There were five residents of psychiatry who performed the interviews with the patients. To calculate the inter-rater reliability, 35 individuals with BD type I were interviewed. All of the five residents attended these interview sessions, where one resident interviewed with the patient and everyone rated the probands independently.

The rate of reliability for the HDRS-7, YMRS, and SAPS scores are shown in the tables 1, 2 and 3.

**Table 1:** The inter-rater reliability of the Hamilton Depressive Rating Scale (HDRS) among five interviewers (n = 35)

Interviewers		A	B	C	D	E
**A**	Pearson correlation	1				
	P value (2 tailed)					
**B**	Pearson correlation	0.936	1			
	P value (2 tailed)	0.001				
**C**	Pearson correlation	0.955	0.924	1		
	P value (2 tailed)	0.001	0.001			
**D**	Pearson correlation	0.959	0.928	0.944	1	
	P value (2 tailed)	0.001	0.001	0.001		
**E**	Pearson correlation	0.939	0.920	0.921	0.930	1
	P value (2 tailed)	0.001	0.001	0.001	0.001	

**Table 2:** The inter-rater reliability of the Young-Mania Rating Scale (Y-MRS) among five interviewers (n = 35)

Interviewers		A	B	C	D	E
**A**	Pearson correlation	1				
	P value (2 tailed)					
**B**	Pearson correlation	0.957	1			
	P value (2 tailed)	0.001				
**C**	Pearson correlation	0.981	0.958	1		
	P value (2 tailed)	0.001	0.001			
**D**	Pearson correlation	0.976	0.933	0.970	1	
	P value (2 tailed)	0.001	0.001	0.001		
**E**	Pearson correlation	0.928	0.874	0.905	0.944	1
	P value (2 tailed)	0.001	0.001	0.001	0.001	

**Table 3:** The inter-rater reliability of the Scale for the Assessment of Positive Symptoms (SAPS) among five interviewers (n = 29)

Interviewers		A	B	C	D	E
**A**	Pearson correlation	1				
	P value (2 tailed)					
**B**	Pearson correlation	0.961	1			
	P value (2 tailed)	0.001				
**C**	Pearson correlation	0.943	0.925	1		
	P value (2 tailed)	0.001	0.001			
**D**	Pearson correlation	0.963	0.918	0.972	1	
	P value (2 tailed)	0.001	0.001	0.001		
**E**	Pearson correlation	0.921	0.878	0.965	0.958	1
	P value (2 tailed)	0.001	0.001	0.001	0.001	

## Discussion

The BDPF is one of the few long-term followup studies investigating the natural course of psychiatric disorders in Iran. In this project, the clinical information about Iranian patients with BD type I and variations in their symptoms with time are gathered. The results could lead to increasing the clinicians’ awareness about the clinical picture of this disorder in Iranian patients, helping the researchers to design future studies, promoting the policy making in mental health in Iran, and providing part of the required information to calculate the burden of the BD type I in Iran. Furthermore, considering the current limited experience in doing prospective longitudinal investigations on patients with mental disorders in Iran, the present study could be useful in recognizing administrative obstacles and solving them during the rest of the present project and for other studies.

Some of the considered inclusion criteria can be seen as methodological limitations because the study sample would not be representative of all of the BD type I patients population referred to this hospital. Actually, in this manner, homeless patients or patients with very low socioeconomic status are excluded; although the individuals referred to this center are not generally from high socioeconomic levels.

## Conclusions

Given generally the lack of any effective and valid system for registering the patients’ clinical data and any continuous after care system in psychiatric centers in Iran, the present project could decrease to some extent the current shortcomings in Iran’s psychiatric data at least about one of the major psychiatric disorders known as the eighth resulting cause in medical disability over the world.^27^
